# Aortic valve planimetry by high-resolution 3-dimensional MR image acquisition with a breath-hold: comparison with conventional cine MR imaging and echocardiography to assess the severity of aortic valve stenosis

**DOI:** 10.1186/1532-429X-18-S1-P342

**Published:** 2016-01-27

**Authors:** Hae Jin Kim, Yeon Hyeon Choe, Sung Mok Kim, Moon Young Kim, Sung-Ji Park

**Affiliations:** 1 grid.414964.a0000000106405613Radiology, Samsung Medical Center, Seoul, Korea (the Republic of); 2 grid.414964.a0000000106405613Cardiology, Samsung Medical Center, Seoul, Korea (the Republic of)

## Background

We intended to evaluate the novel application of high-resolution 3-dimensional MR image acquisition with single-breath-hold SSFP sequence to calculate the aortic valve area (AVA).

## Methods

In 88 consecutive patients (66.9 ± 9.59 years, 63% men) with varying degrees of aortic valve stenosis, high-resolution 3D SSFP images (3D planimetry; 2.0 mm slice thickness, 20 contiguous slices; image matrix, 256 ' 209) were acquired with single breath-hold during mid systole and mid diastole. SSFP cine MR imaging (2D planimetry) and velocity-encoded cine MRI (slice thickness, 4.5 mm) in three levels of aortic valve were also performed. AVA area was measured by two experienced observers using commercial software (iNtuition, TeraRecon). MR imaging measurements and image quality were compared with transthoracic echocardiographic measurements of effective aortic orifices (EAO) using the continuity equation (1 = severe blurring of images, 2 = moderate blurring of valve contours; 3 = mild blurring of valve contours, 4 = excellent and no artifact). Sensitivity for accurate measurement and receiver operating characteristic (ROC) curve were calculated. Intra- and interobserver agreements were determined by using intraclass correlation coefficient (ICC).

## Results

Mean AVA derived by 3D planimetry, 2D planimetry, and echocardiography were 0.77 ± 1.04 cm^2^, 0.72 ± 1.16 cm^2^, and 0.75 ± 0.32 cm^2^, respectively. The ICC value of 3D planimetry was higher than 2D planimetry [0.799 (CI, 0.691-0.869) vs. 0.743 (CI, 0.605-0.832)] with echocardiographic EAO as the standard of reference. The grade of image quality of 3D planimetry was superior to 2D planimetry (3.65 ± 0.65 vs. 3.17 ± 0.65). The correlation coefficients of maximum peak velocity on velocity-encoded cine MR imaging with 3D planimetry and that with 2D planimetry were 0.42 (p < 0.05) and 0.35 (p < 0.05). Intra- and interobserver agreements for 3D planimetry were excellent [ICC = 0.949 (CI, 881-979) and 0.846 (CI, 0.636-0.935), respectively; both, p = 0.000).

## Conclusions

Novel application of high-resolution 3D SSFP breath-hold MR imaging enables planimetry of AVA in patients with valvular aortic stenosis with better image quality than 2D planimetry with conventional cine MR imaging.

